# Association between anogenital distance as a noninvasive index in the diagnosis and prognosis of reproductive disorder: A systematic review

**DOI:** 10.18502/ijrm.v21i8.14016

**Published:** 2023-09-20

**Authors:** Parisa Zamani, Zeinab Hemati, Roya Kelishadi, Sakineh Kolahdozan, Mostafa Dianatinasab, Mojtaba Keikha

**Affiliations:** ^1^Student Research Committee, School of Nursing and Midwifery, Shahroud University of Medical Sciences, Shahroud, Iran.; ^2^Child Growth and Development Research Center, Research Institute for Primordial Prevention of Non-Communicable Disease, Isfahan University of Medical Sciences, Isfahan, Iran.; ^3^Clinical Research Development Unit, Bahar Hospital, Shahroud University of Medical Sciences, Shahroud, Iran.; ^4^Department of Medical Sciences, School of Medical and Life Sciences, Sunway University, Malaysia.; ^5^Department of Complex Genetics and Epidemiology, School of Nutrition and Translational Research in Metabolism, Maastricht University, Maastricht, The Netherlands.; ^6^Department of Biostatistics and Epidemiology, Faculty of Public Health, Kerman University of Medical Sciences, Kerman, Iran.

**Keywords:** Genitalia, Prognosis, Early diagnosis, Reproductive health

## Abstract

**Background:**

There are 2 measures of anogenital distance (AGD) in men and women. AGD has been used as an indicator of fetal androgen dysfunction and an adverse outcome in adulthood. Some studies have shown the association of AGD as a predictor in the diagnosis and prognosis of diseases and disorders.

**Objective:**

To systematically summarize the latest evidence for presenting AGD as a new approach for prognosis and early diagnosis of diseases.

**Materials and Methods:**

A systematic review of the available literature was performed using Medline via PubMed, Scopus, and ISI Web of Knowledge up to July 2021, using search terms “anogenital distance" OR “anogenital index" OR “ano genital distance" OR “ano genital index". Language restrictions were not imposed.

**Results:**

After reviewing the retrieved articles, 47 unique studies were included in this systematic review. Different outcomes, including endometriosis, prostate cancer, polycystic ovary syndrome, pelvic organ prolapse, hypospadias, cryptorchidism, fertility and semen parameters, maternal and birth development, and ovarian and gynecological-related disorders, have been studied in the included evidence. A negative association was observed between AGD and endometriosis and hypospadias and a positive association between AGD and prostate cancer, polycystic ovary syndrome, male fetal gender, and fertility parameters.

**Conclusion:**

Using quantitative indicators such as AGD may be a useful clinical tool for the diagnosis of diseases. Although many studies have shown an association between AGD and diseases, some factors, including different measurement methods, different measurement tools, age, and different definitions of AGD, can be involved in the variation of AGD.

## 1. Introduction

Several lifestyle factors affect human reproductive performance (1). Exposure to endocrine-disrupting chemicals in fetal life can be concerning because the sexual organs are formed in this period, which is the foundation of reproductive health in adulthood (2, 3). Reproductive disorders and anogenital problems can be debilitating and negatively impact people's quality of life (4, 5). In the past decades, reproductive disorders and anogenital problems have increased dramatically (6-8).

Diagnostic procedures may generally be associated with health-related complications (9, 10). Some other methods result in high financial costs and time for the individuals and delay the “golden time” for diagnosis and treatment of the disease (11). Therefore, a noninvasive indicator that predicts and diagnoses the disease earlier seems important.

Hormonal dysfunction during fetal life can cause anogenital problems in adulthood (12-14). As a result, we can use anogenital distance (AGD) as a proxy for hormonal dysfunction in the past to determine whether or not a hormonally disruptive action occurred during fetal growth (15, 16). AGD is a noninvasive and easily measurable anthropometric measurement, the distance between the anus and the genital tubercle (17-20). It is considered the amount of distance between the anterior clitoral surface to the upper verge of the anus (AGD ${}_{\text{AC}}$ ) and distance between posterior fourchette to the upper verge of the anus in women (AGD ${}_{\text{AF}}$ ) (21). It is indicated the amount of distance between cephalad insertion of the penis to the center of the anus (AGD ${}_{\text{AP}}$ ) and distance between posterior base of the scrotum to the center of the anus in male (AGD ${}_{\text{AS}}$ ) (22).

AGD has been used as an indicator of fetal androgen dysfunction and as an adverse outcome in adulthood (23). AGD is a broad marker that retrospectively describes fetal androgen disruption and potentially adult reproductive disorders (24). Intrauterine hormonal changes can affect AGD (25, 26). Thus, AGD is a biomarker of prenatal hormonal exposure at birth and can also reflect reproductive health in adulthood (12, 27). In men, correlations have been found between the length of AGD and semen quality, testicular volume, hypospadias, and cryptorchidism (28-31). Also, in women, AGD is a potential determinant of some female gynecologic and reproductive disorders, such as polycystic ovary syndrome (PCOS), endometriosis, and pelvic organ prolapse (POP) (31-33). AGD can also determine the gender of the fetus in the first trimester of pregnancy (11).

There are large numbers of studies supporting the ability of AGD as a tool for disorder prediction. Thus, in this study, we have systematically summarized the available evidence related to assessing the association of AGD in the diagnosis and prediction of different health conditions.

## 2. Materials and Methods

### Literature search

The current systematic review was performed according to preferred reporting items for systematic reviews and meta-analyses (PRISMA) statement (34). The electronic databases, including Medline via PubMed, Scopus, and ISI Web of Knowledge up to July 30, 2021 were systematically searched. The following keywords were used: “anogenital distance" OR “anogenital index" OR “ano genital distance" OR “ano genital index". Boolean Operators (AND, OR) were used in search strategy. Also, we review the reference of all included paper for any related articles. No any language limitation considered in the search strategy.

### Selection criteria

The below criteria were used for selecting studies.

Inclusion criteria:

1. All quantitative research evaluated the association of AGD in diagnosing or prognosis of any disorder or disease.

2. Studies that have a proper definition of AGD.

3. All observational and comparative studies such as cross-sectional, case-control, and cohort studies.

Exclusion criteria:

1. All studies that are not the original studies such as letter, editorial.

2. Studies conducted on animals.

3. Laboratory and in vivo studies.

### Data management

For removing the duplicate references and managing retrieved evidences we used EndNote software.

### Data extraction and abstraction

**Figure 1:**
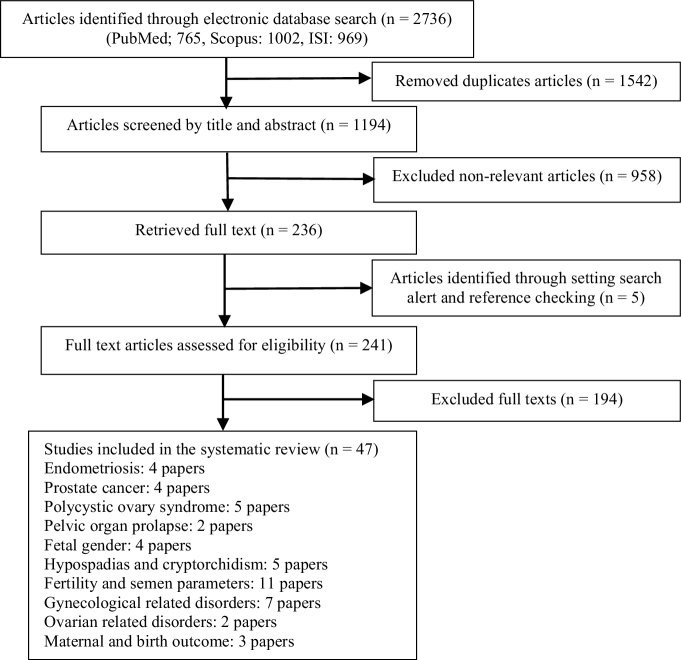
Papers search and review flowchart for selection of primary study.

As presented in the PRISMA flowchart, we retrieved 1194 unique references after removing the duplicates. In total, 1542 articles were duplicated in basic search, found, and removed by the EndNote software. Another 958 were excluded after the title and abstract review. The full texts of the remaining 241 articles were retrieved and critically evaluated. This systematic review comprised 47 publications after the screening procedure (Figure 1).

2 independent reviewers (MK and PZ) reviewed the full text of publications identified by the literature search for their possible relevance or screened the titles and abstracts for inclusion in the review.

If there was a dispute, it was settled by consulting with a third reviewer (SK). The data was abstracted separately by 2 reviewers (ZH and MD). The following information was retrieved from all eligible papers: first author's name, year of publication, study location, age group, participant characteristics, study design, type of outcome, type of AGD, and key findings. According to high heterogeneity between included studies quantitative meta-analysis was not performed.

### Quality assessment

The Newcastle-Ottawa Scale was used for assessing the quality of included studies (35). The Newcastle-Ottawa Scale score have 9 score and 3 main criteria. Each investigation was assessed based on 3 main criteria: 1) appropriate study population selection, 2) comparability of study groups, and 3) determination of the desired exposure (in cohort studies) or result (in case-control studies).

Each publication was evaluated independently by 2 reviewers. Disagreements were worked out via conversation until a consensus was reached. Studies with a score of 7 or more out of 9 were considered to be of high quality. Table I displays the results of each study's quality evaluation.

**Table 1:** Classification of the result according to each category of outcomes


**Authors, Year,** **Location (Ref)**	**Direction**	**Characteristics of participants**	**Age group**	**Type of outcome**	**Type of AGD**	**Main findings**	**NOS ${}^{+}$ score**
**Endometriosis**							
**Sanchez-Ferrer ** ***et al.*** **,** **2019, Spain (36) ${}^{\text{a}}$ **	↓ AF	Subjects with endometriosis and healthy group	18-50 yr	Endometriosis	AF, AC	Female with endometriosis had significantly shorter AGD ${}_{\text{AF}}$ (22.8 ± 4.6 s 27.2 ± 5.7 mm; p < 0.001)	5
**Mendiola, ** ***et al.*** **, 2016,** **Spain (21) ${}^{\text{a}}$ **	↓ AF	Women with or without endometriosis (endometriomas and/or deep endometriosis)	18-50 yr	Endometrioma and deep endometriosis	AF, AC	AGD ${}_{\text{AF}}$ , was related to endometriomas/ deep endometriosis (p < 0.001-0.04), but AGD ${}_{\text{AC}}$ not related	6
**Sanchez-Ferrer ** ***et al.*** **,** **2017, Spain (37)** ** ${}^{\text{a}}$ **	↓ AF	Premenopausal women	18-50 yr	Endometriosis	AF, AC	The AGD ${}_{\text{AF}}$ , but not AGD ${}_{\text{AC}}$ was related with the endometriomas, but AGD ${}_{\text{AC}}$ not related	3
**Crestani ** ***et al.*** **, 2020,** **France (38)** ** ${}^{\text{b}}$ **	↓ AF ↓ AC	Subjects who selected for pelvic surgery	34.1 ± 6.6 in the case and 39.9 ± 9.3 in the control group	Endometriosis	AF, AC	The presence of endometriosis was negatively associated with both the AGD ${}_{\text{AF}}$ (β = -9.66 mm [CI-12.20 to -7.12]) and AGD ${}_{\text{AC}}$ (β = -13.75 mm [CI-19.37 to -8.12]) in multivariable analysis. AGD ${}_{\text{AF}}$ had a better predictive index than AGD ${}_{\text{AC}}$ for perceptive the presence of endometriosis with an AUC of 0.840 and 0.756	9
**Prostate Cancer**							
**Maldonado-Carceles** ***et al.*** **, 2017, Spain (39)** ** ${}^{\text{c}}$ **	↑ AS	Prostate cancer individuals	66.9 ± 6.5	Prostate cancer	AS, AP	In men: the quantity of highest gleason score was related with longer AGD ${}_{\text{AS}}$ (p = 0.015) but not for AGD ${}_{\text{AP}}$	5
**Onate-Celdran ** ***et al.*** **, ** **2019, Spain (40) ${}^{\text{a}}$ **	↑ AS ↑ AP	Men in a hospital outpatient clinic	Cases: 61.8 ± 5.6 Controls: 50.2 ± 12.3	Prostate cancer	AS, AP	AGD ${}_{\text{AS}}$ and AGD ${}_{\text{AP}}$ were significantly shorter in the control group	6
**Castaño-Vinyals ** ***et al.*** **,** **2012, Spain (41) ${}^{\text{a}}$ **	↓ AP	Cases were consecutive individuals with subjects with prostate cancer, and control group from urology outpatient departments	65 ± 7	Prostate cancer	AS, AP	A higher AGD ${}_{\text{AP}}$ was related with a lower OR for prostate cancer (OR per 5 mm increase in AGD ${}_{\text{AP}}$ , 0.83; 95% CI, 0.70-0.99; p = 0.03). This association was not observed for AGD ${}_{\text{AS}}$ (OR per 5 mm variation, 0.96; 95% CI, 0.82-1.13)	3
**Sahin, 2019 ** ***et al.*** **,** **Turkey (42) ${}^{\text{a}}$ **	↑ AP	Individual with prostate cancer and subjects with prostatic hyperplasia in benign form	Cases: 67.7 ± 7.74 Controls: 67.03 ± 7.89	Prostate cancer	AS, AP	AGD ${}_{\text{AP}}$ in patients with prostate cancer was statistically longer than in those diagnosed with benign prostatic hyperplasia (p = 0.000), but not for AGD ${}_{\text{AS}}$ (p = 0.823)	5
**Polycystic ovary syndrome**							
**Hernandez-Penalver** ***et al.*** **, 2018, Spain (43)** ** ${}^{\text{a}}$ **	↑ AC	Females admitted in the gynecology unit of the hospital	18-40 yr	Polycystic ovary syndrome	AF, AC	AGD ${}_{\text{AC}}$ , but not AGD ${}_{\text{AF}}$ , was related with PCOS (p < 0.001 to 0.048). AGD ${}_{\text{AC}}$ was larger in PCOS compared with controls group	7
**Barrett ** ***et al.*** **, 2016,** **USA (44) ${}^{\text{c}}$ **	↑ AF	Child girls born to women with PCOS	Mothers: 18 or older	Polycystic ovary syndrome	AF, AC	AGD was higher in the daughters of women with a PCOS diagnosis than the child of women with no PCOS (AGD ${}_{\text{AF}}$ : β = 1.21, p = 0.05; AGD ${}_{\text{AC}}$ : β = 1.05, p = 0.18)	7
**Sanchez-Ferrer ** ***et al.*** **,** **2017 Spain (32)** ** ${}^{\text{a}}$ **	↑ AC	Females admitted the gynecology unit of the hospital with or without PCOS	18-40 yr	Polycystic ovary syndrome	AF, AC	AGD ${}_{\text{AC}}$ , but not AGD ${}_{\text{AF}}$ , was related with the patients with PCOS (p = 0.002-0.008). Females with AGD ${}_{\text{AC}}$ in the upper compared to the lowest tertile were 2.9-times (95% CI, 1.4-5.9; P-trend = 0.008) more possible to have PCOS	7
**Simsir ** ***et al.*** **, 2019,** **Turkey (45) ${}^{\text{b}}$ **	↑ AC ↑ AF	Females with PCOS and women in healthy controls group	18-40 yr	Polycystic ovary syndrome	AF, AC	AGD ${}_{\text{AC}}$ and AGD ${}_{\text{AF}}$ were both higher in the PCOS group. The mean values of ratio ant/post were 4.4 ± 1.0 and 4.9 ± 1.0 in the PCOS and healthy groups, respectively (p = 0.003)	9
**Wu ** ***et al.*** **, 2017, ** **China (31)** ** ${}^{\text{a}}$ **	↑ AC ↑ AF	Females with PCOS admitted in out- patient department of gynecology, and controls were healthy women who underwent routine tests	18-35 yr	Polycystic ovary syndrome	AF, AC	Subjects with AGD ${}_{\text{AF}}$ in the highest tertile were 18.8 times (95% CI, 9.6-36.6; p < 0.001) more probable to have PCOS than those in the lowest tertile. Women with AGD ${}_{\text{AC}}$ in the longer tertile were 6.7 times (95% CI, 3.7-12.1; p < 0.001) more possible to have PCOS than those in the lowest tertile	8
**POP**							
**Sanchez-Ferrer ** ***et al.*** **,** **2016, Spain (29) ${}^{\text{a}}$ **	↓ AF ↑ AC	Individuals who had gynecology consultation	Cases: 65.1 ± 9 Controls: 50 ± 7.7	POP	AF, AC	The cases had a significantly shorter AGD ${}_{\text{AF}}$ than the control individual (p = 0.001) and a considerably longer AGD ${}_{\text{AC}}$ than the control individual (p = 0.0001)	6
**Sanchez-Ferrer ** ***et al.*** **,** **2018, Spain (46) ${}^{\text{a}}$ **	↓ AF ↑ AC	Females over 40 yr of age, who had seeking care for genital lumps	> 40 yr	POP	AF, AC	There were significant differences between POP subjects and the controls for AGD ${}_{\text{AC}}$ (88.1 ± 19.7 mm vs. 70.1 ± 11.7 mm, p = 0.0001) and AGD ${}_{\text{AF}}$ (18 ± 5.4 mm in POP vs. 23 ± 5 mm in controls, p = 0.001). AGD ${}_{\text{AF}}$ was smaller in women with prolapse, but AGD ${}_{\text{AC}}$ was higher in women with POP	5
**Fetal gender**							
**Arfi ** ***et al.*** **, 2016, France (47)** ** ${}^{\text{b}}$ **	↑ M	Pregnant women at 11-14 wk of gestation	11-14 wk of gestation	Fetal gender	AGD	Gender was accurately determined for 87% of the males and 89% of the females. The AGD of the male fetuses was longer than for female fetuses (mean value 6 mm [IC95% 5.8-6.2] vs. 4.2 mm [IC95% 4-4.3], p < 0.0001)	6
**Fowler ** ***et al.*** **, 2016, New Zealand **(48) ** ${}^{\text{c}}$ **	↑ M	Normal fetuses	11-12 wk of gestation	Fetal smoke exposure and sex differences	APP	All AGD (AGD ${}_{\text{APP}}$ ) measures were signiﬁcantly (p = 0.05-0.001) lower in females than males	7
**Najdi ** ***et al.*** **, 2019, Iran (49)** ** ${}^{\text{c}}$ **	↑ M	Females aged 18-35 yr old with a single pregnancy	11-13 wk and 6 days of gestation	Fetal gender	AGD	The average AGD of the females was significantly shorter than that of the males	7
**Sipahi ** ***et al.*** **, 2019, Turkey (11)** ** ${}^{\text{c}}$ **	↑ M	Females with a single pregnancy from 11-13 wk	11-13 wk and 6 days of gestation	Fetal gender	AGD	The likely of being a female was 100% when an AGD < 4.8 mm was diagnosed with ultrasound, and the likely of being a male was 90% when an AGD of 4.8 mm was diagnose using ultrasound	6
**Hypospadias and cryptorchidism**							
**Cox ** ***et al.*** **, 2017, UK (50)** ** ${}^{\text{a}}$ **	↓ AS ↓ AP	Cases were boys undergoing hypospadias surgery, and controls were healthy boys undergoing circumsision in the operation theatre	< 2 yr	Hypospadias	AS, AP	The median control AGD ${}_{\text{AP}}$ was 74 mm, and for boys, with hypospadias, it was 71.29 mm. The median control AGD ${}_{\text{AS}}$ was 42.3 mm and 39.37 mm in hypospadias. Both AGD ${}_{\text{AP}}$ and AGD ${}_{\text{AS}}$ were significantly shorter in the case group than in the control group	5
**Gilboa ** ***et al.*** **, 2017, Israel (30)** ** ${}^{\text{c}}$ **	↓ AS	Male fetuses with suspected genital abnormalities	Hypospadias and buried penis	AS	A signiﬁcant difference was showed between the normal mean AGD for gestational age compare with hypospadias (mean: 6 SD, 16.9064.08, and 11.6863.31 mm, respectively). However, no significant difference was showed between the normal mean AGD for gestational age compare with a buried penis (18.8562.76 and 19.4663.41 mm)	5
**Hsieh ** ***et al.*** **, 2008, USA (51)** ** ${}^{\text{c}}$ **	↓ AS	Boys undergoing urologic procedures	4-86 months	Hypospadias	APP	Boys with hypospadias had shorter AGD than that of boys with normal genitals (67+1.2 vs. 73+1 mm respectively, p = 0.002)	7
**Hypospadias and cryptorchidism**							
**Singal ** ***et al.*** **, 2016, India (52)** ** ${}^{\text{c}}$ **	↓ AS	Pre-pubertal boys	5 months to 14 yr of age	Hypospadias	AGD ${}_{\text{AS}}$ , AGD ${}_{1}$ and AGD ${}_{2}$	Of the 3 AGDs scales, we found only AGD ${}_{\text{AS}}$ to be signiﬁcantly lower in boys with hypospadias (40.6+9.7 mm vs. 45.6+9.4 mm, p < 0.001). A signiﬁcant negative association was seen with all the scales of AGD's with the severity of hypospadias	7
**Thankamony ** ***et al.*** **, 2014, UK (53)** ** ${}^{\text{a}}$ **	↓ AS	Boys with isolated hypospadias or cryptorchidism	< 2 yr	Hypospadias or cryptorchidism	AS	Boys with hypospadias had lower AGD than healthy boys (p < 0.0001). AGD in boys with cryptorchidism was longer than in boys with hypospadias (p < 0.01) and shorter than AGD in healthy boys (p < 0.0001)	6
**Fertility and semen parameters**							
**Eisenberg ** ***et al.*** **, 2015, USA (54)** ** ${}^{\text{b}}$ **	↑ AS	“Men with a history of infertility, sexual dysfunction, hypogonadism, fecundity anxiety, or vasectomy, aged 18 or older”	> 18 yr	Azoospermia	AS	Men with obstructive azoospermia had signiﬁcantly smaller AGD than those with nonobstructive azoospermia (mean: 41.9 vs. 36.3 mm; median 40 vs. 31.2 mm; p = 0.01)	7
**Freire ** ***et al.*** **, 2018, Spain (28)** ** ${}^{\text{c}}$ **	↑ AS	“9-11-yr-old boys”	9-11 yr	Reproductive outcomes	AS	AGD was positively associated with testicular size, with a 6% increase in the odds of increased testicular volume ( > 3 mL) for each 10% rise in AGD (OR = 1.06, 95% CI = 1.00-1.19). In contrast, no signiﬁcant association was showed between AGD and tanner stage, cryptorchidism, or serum hormone levels	9
**Mendiola ** ***et al.*** **, 2015, Spain (55)** ** ${}^{\text{c}}$ **	↑ AS	Male partners of infertile couples admitted in a semen analysis clinic	23-48 yr	Semen quality	AS, AP	Signiﬁcant positive relationship between AGD ${}_{\text{AS}}$ scales and sperm concentration, total sperm count, and total sperm motile count were showed (p < 0.05). No other AGD scales were signiﬁcantly associated with any other sperm parameters	9
**Mendiola ** ***et al.*** **, 2011, USA (56)** ** ${}^{\text{c}}$ **	↑ AS	126 volunteer men in Rochester, New York	18-22 yr	Semen quality	AS, AP	AGD ${}_{\text{AS}}$ was positively associated with sperm concentration, motility, morphology, total sperm count, and total motile count (p = 0.002, 0.028, 0.048, 0.006, and 0.009, respectively). The risk of subfertility was rised 7.3 times (95% CI, 2.5-21.6) for an (adjusted) AGD ${}_{\text{AS}}$ below the median compared with AGD ${}_{\text{AS}}$ above the median	10
**Fertility and semen parameters**							
**Mira-Escolano ** ***et al.*** **, 2014, Spain (57)** ** ${}^{\text{c}}$ **	↑ AF	Young college students	18-23 yr	Reproductive hormone levels	AF, AC	AGD ${}_{\text{AF}}$ was positively associated with serum testosterone levels (95% CI, 0.01, 0.10; p = 0.02). None of the measurements was associated with other reproductive hormones	9
**Eisenberg ** ***et al.*** **, 2015, USA (54)** ** ${}^{\text{b}}$ **	↑ AS	Men at a urology clinic	43 ± 13 yr	Male fertility	AS	AGD was significantly greater in men with higher sperm concentration, total sperm count, and total motile sperm count	7
**Zhou ** ***et al.*** **, 2016, China (24)** ** ${}^{\text{b}}$ **	- Young college students	20.1 ± 1.6 yr	Semen parameters	AS, AP	“Both AGD ${}_{\text{AS}}$ and AGD ${}_{\text{AP}}$ were not associated with any semen parameters. AGD ${}_{\text{AP}}$ was correlated with sperm progressive motility and reproductive hormones of E2, testosterone, SHBG, and the testosterone/LH ratio. AGD ${}_{\text{AP}}$ was negatively associated with the serum E2 level (95% CI, 20.198 to 20.043; p = 0.002) and positively related to the ratio of T/E2 (95% CI, 0.004-0.011; p = 0.001)”	6
**Parra ** ***et al.*** **, 2016, Spain (22)** ** ${}^{\text{b}}$ **	- University students	18-23 yr	Semen quality	AS, AP	AGD scales were not associated with any semen parameters or any of the reproductive hormone levels	5
**Eisenberg ** ***et al.*** **, 2011, USA (58)** ** ${}^{\text{c}}$ **	- Men being evaluated for infertility and men with proven fertility	Cases: 35.3 ± 17.4 Controls: 44.8 ± 9.7	Male fertility and semen parameters	AS	The infertile men showed significantly smaller mean AGD than the fertile controls (31.8 vs. 44.6 mm). AGD was significantly related with sperm density (95% CI, 0.53, 8.09, p = 0.03) and total motile sperm count (95% CI, 1.34, 10.58, p = 0.01)	6
**Glintborg ** ***et al.*** **, 2019, Denmark (59)** ** ${}^{\text{b}}$ **	↑ AS ↑ AP	Pregnant women at gestational week 28 and their children at the age of 3 months	30 yr	Testosterone levels	In girls: AF, AC in boys: AS, AP	Maternal testosterone levels were positively related with AGD ${}_{\text{AS}}$ and AGD ${}_{\text{AP}}$ in boys, whereas AGD scales in girls were not correlated to maternal testosterone levels	7
**Wainstock ** ***et al.*** **, 2017, Israel (60)** ** ${}^{\text{c}}$ **	- Pregnant women during the first stage of labor	26.69 ± 5.26	Fertility	AF	AGD was positively related with maternal age (B = 0.032, 95% CI, 0.007-0.05, p = 0.01) and it was negatively related with infertility treatments (B = -1.06, 95% CI, -1.99 to -0.12, p = 0.03). AGD was not related with parity, ethnicity, height, or vaginal deliveries	9
**Gynecological related disorders**							
**Moya-Jiménez ** ***et al.*** **, 2018, Spain (61)** ** ${}^{\text{b}}$ **	↓ AC	Participants with vaginal deliveries	Case: 33.5 ± 5.5 Controls: 30.4 ± 6.1	Episiotomy	AF, AC	AGD ${}_{\text{AC}}$ was significantly smaller in the episiotomy group. Adjusted mean values for AGD ${}_{\text{AC}}$ in cases and healthy group were 92.4 vs. 98.0 mm, respectively	7
**Eisenberg ** ***et al.*** **, 2012, USA (62)** ** ${}^{\text{c}}$ **	- Men with varicocele	33.1 ± 6.3	Efﬁcacy of varicocele repair	AS	Men with longer AGDs showed improvements in total motile sperm count and sperm concentration after varicocelectomy than men with shorter AGDs. In contrast, there were no signiﬁcant relation in improvements in semen volume or sperm motility between men based on AGD heigh	5
**Jain ** ***et al.*** **, 2013, India (63)** ** ${}^{\text{c}}$ **	↓ AS	Born male infants	Within the first 48 hr after birth	Undescended testis	AGI, AS	AGD was signiﬁcantly smaller in child with UDT when compared with infants with descended testis (mean ± SD; 2.21 ± 0.36 vs. 2.56 ± 0.31 cm; p < 0.001). AGD was also signiﬁcantly lower in infants with UDT (mean ± SD; 1.68 + 0.27 vs. 1.81 ± 0.20 cm/kg; p < 0.001)	9
**Domenici ** ***et al.*** **, 2018, Italy (64)** ** ${}^{\text{a}}$ **	↓ AF	Premenopausal and postmenopausal women	Cases: 45-80 yr Controls: 20-45 yr	Vulvo-vaginal Atrophy	AF, AGI	“AGD (30.87 ± 2.98 vs. 17.57 ± 2.18; p = 0.0001) and AGI (1.40 ± 0.21 vs. 0.70 ± 0.15; p = 0.0001) were both significantly lower in the postmenopausal group”	6
**Wainstock ** ***et al.*** **, 2019, Israel (12)** ** ${}^{\text{b}}$ **	↓ AF	Parturients with singleton, term, and cephalic presentation	33-59 yr	Gynecological or any of the other morbidity categories, including cardiovascular morbidities	AF, AS	“The rate of encounters due to gynecological conditions was significantly higher among women with below mean AGD than the above mean AGD group (36.6% vs. 23.4%, p = 0.03). Rates of all other health categories encounters were not significantly different between the 2 study groups, including incidence rates of cardiovascular-related encounters (16.6% vs. 16.8% among the below vs. above AGD groups, OR = 1.02; 0.54-1.92, p = 1.0)”	7
**Toprak ** ***et al.*** **, 2020, Turkey (65)** ** ${}^{\text{c}}$ **	- Men with from the beginning of life premature ejaculation and men without any problem	37 ± 7.89 yr	Premature ejaculation	AS, AP	A significant association was detected between AGD ${}_{\text{AS}}$ and premature ejaculation (r = 0.199, p = 0.019). However, no statistically significant diversity were showed between AGD ${}_{\text{AP}}$ and premature ejaculation	8
**Gynecological related disorders**							
**Sertkaya ** ***et al.*** **, 2020, Turkey (66)** ** ${}^{\text{a}}$ **	↓ AS ↓ AP	Premature ejaculation group and control group	30.73 ± 4.40 yr	Premature ejaculation	AS, AP	In the cases group, AGD ${}_{\text{AP}}$ and AGD ${}_{\text{AS}}$ were found to be lower (77.46 ± 2.31 and 54.78 ± 2.56 mm) than in the healthy group (81.32 ± 3.11 and 58.16 ± 3.48 mm)	6
**Ovarian related disorders**							
**Fabregues ** ***et al.*** **, 2018, Spain (33)** ** ${}^{\text{b}}$ **	↓ AC ↓ AF	Women undergoing controlled ovarian stimulation for IVF	Poor responders = 37.9 ± 0.9 norm responders = 36.8 ± 0.4 high responders = 36.1 ± 1.5	Ovarian response	AF, AC	Smaller AGD ${}_{\text{AC}}$ and AGD ${}_{\text{AF}}$ were correlated with poor ovarian response (p < 0.001). Both AGD ${}_{\text{AC}}$ and AGD ${}_{\text{AF}}$ presented a positive and signiﬁcant correlation with the total number of oocytes retrieved (r = 0.29 and r = 0.28, respectively; p < 0.05)	6
**Mendiola ** ***et al.*** **, 2012, Spain (67)** ** ${}^{\text{c}}$ **	↑ AC ↑ AF	College students	20 ± 1.2	Ovarian follicular number	AF, AC	Both AGD scales were positively related with ovarian follicle number, with AGD ${}_{\text{AF}}$ being more strongly correlated	10
**Maternal and birth outcome**							
**Park ** ***et al.*** **, 2015, Korea (68)** ** ${}^{\text{c}}$ **	↓ III	Newborn male infants	Newborn	Birth weight	I, II, III	AGDI was significantly lower in the low-birth weight group than in the healthy group (p < 0.001)	9
**Liu ** ***et al.*** **, 2015, China (69)** ** ${}^{\text{b}}$ **	- Healthy pregnant women who delivered in a local hospital	Maternal age in the male neonate: 27.06 ± 4.52 maternal age in the female neonate: 27.12 ± 4.21	Thyroid hormone status (TSH, FT4, FT3) in umbilical cord serum	AS, AF	Higher AGD in male newborns was observed with greater cord serum FT3 (95% CI, 0.58-2.13), FT4 (95% CI, 0.00-0.25), TSH (95% CI, 0.65-5.63), and lower FT4/FT3 ratio (95% CI, -0.20- -0.02). The association between AGD and THs was not statistically significant, in female neonates	7
**Kumar Singal ** ***et al.*** **, 2016, India (70)** ** ${}^{\text{c}}$ **	- Newborns born at a secondary-level district hospital	the ﬁrst 48 hr of birth	“Maternal (age at the time of conception, gravidity, and parity) and infant characteristics (birth weight, length, and gestational age)”	AS, AF	“Only birth weight (b = 0.229, 95% CI, 0.150-0.308, p < 0.001) and gestational age (b = 0.029, 95% CI, 0.010-0.048, p = 0.003) were statistically signiﬁcant predictors of AGD in males. For female infants, birth weight (b = 0.135, 95% CI, 0.095-0.175, p < 0.001), gestational age (b = 0.015, 95% CI, 0.007-0.023, p < 0.001), and length (b = 0.008, 95% CI, 0.001-0.015, p = 0.023) were found to be statistically signiﬁcant predictors for AGD. No signiﬁcant association was observed with AGD in boys or girls for gravidity, parity, or maternal age”	7
a: Case-control, b: Cohort, c: Cross-sectional. AGD: Anogenital distance, NOS: The Newcastle-Ottawa Scale, AGD ${}_{\text{AF}}$ : Distance from the posterior fourchette to the upper verge of the anus in males, AGD ${}_{\text{AC}}$ : Distance from the anterior clitoral surface to the upper verge of the anus in women, AUC: Area Under Curve, AGD ${}_{\text{AP}}$ : From the cephalad insertion of the penis to the center of the anus in males, AGD ${}_{\text{AS}}$ : From the posterior base of the scrotum to the center of the anus in women, POP: Pelvic organ prolapse, M: Male, AGD ${}_{\text{APP}}$ : Centre of the anus to the posterior/caudal root of penis/clitoris, AGD1: From the midpoint of the anus to the anterior base of the penis, AGD2: From the midpoint of the anus to the posterior base of the penis, E2: Estradiol, SHBG: Sex hormone-binding globulin, LH: Luteinizing hormone, T: Testosterone, UDT: Un descended testis, AGDI: Distance from the anterior aspect of the penis to the anal verge, AGDII: Distance from the posterior aspect of the penis to the anal verge, AGDIII: Distance from the posterior aspect of the scrotum to the anal verge, TSH: Thyroid stimulating hormone, FT: Free T4, THs: Thyroid hormones							

### Ethical considerations

The present study was approved by Shahroud University of Medical Sciences Ethical Committee, Shahroud, Iran (Code: IR.SHMU.REC.1398.140).

## 3. Results

In this systematic review, we studied the association of AGD as a surrogate for diagnosing different diseases. Different outcomes have been studied, including endometriosis, prostate cancer, PCOS, POP, hypospadias, and cryptorchidism, fertility and semen parameters, maternal and birth outcomes, and ovarian and gynecological disorders related to pregnancy. The results of the included research are summarized in table I. We classified the results according to each outcome category.

Overall, a negative association was observed between AGD, endometriosis, and hypospadias, and a positive association between AGD and prostate cancer, PCOS, male fetal gender, and fertility parameters.

### Quality assessments of studies

The methodological quality of the included articles according to NOS is provided in the supplementary file. Also, the overall NOS scores for the included studies are shown in table I. As shown, 22 high-quality and 12 medium-quality studies were included.

This is the first systematic review to assess the association of AGD as a non-invasive alternative to diagnostic and prognostic diseases requiring clinical intervention.

We did a comprehensive, systematic search of the literature to find studies that investigated the association of AGD as a non-invasive alternative to diagnostic and prognostic diseases. Important procedures, including searching, data extraction, and quality assessment, were also carried out independently by 2 experts.

### Implications for clinical practice

Using quantitative indicators such as AGD to determine the prognosis and early diagnosis of diseases in the future may be of great help in therapeutic interventions and the treatment of individuals in the early stages of the disease.

### AGD measurements and endometriosis 

The current evidence showed that a shorter AGD was significantly associated with endometriosis. Few studies exist about AGD in women. Previous studies have shown that AGD was longer in women with a higher ovarian follicular number and higher testosterone levels (71), and disorder in the menstrual cycle before pregnancy (57). One study showed a strong relationship between endometriosis and shorter AGD ${}_{\text{AF}}$ (21). A case-control study of 114 women with endometriosis and 105 controls revealed that shorter AGD was seen in women with endometriosis (36). A prospective cohort study among 168 women over 18 yr old showed that the diagnosis of endometriosis was negatively associated with both the AGD ${}_{\text{AF}}$ and the AGD ${}_{\text{AC}}$ , and the AGD ${}_{\text{AF}}$ had a better predictive value than the AGD ${}_{\text{AC}}$ for discriminating the presence of endometriosis (38). Another study suggests that AGD biomarkers may be useful in diagnosing endometriosis in women (37). AGD is a bidirectional marker that is shorter in women with exposure to estrogens, for example, endometriosis, and in women with exposure to antiandrogens, such as phthalates (72). On the other hand, AGD is longer in a woman with relatively high levels of androgens, like PCOS. Therefore, AGD is a biomarker that may be useful in identifying the intrauterine environment from the prenatal period to adulthood (73).

### AGD measurements and prostate cancer 

Based on the included evidence, a correlation was observed between AGD and prostate cancer. One study demonstrated that AGD ${}_{\text{AP}}$ was higher in individuals with prostate cancer than in cases of prostatic hyperplasia (42). Another cross-sectional study among 120 prostate cancer patients showed that AGD ${}_{\text{AS}}$ was positively associated with the highest Gleason score and D'Amico nomogram (39). A previous study in 60 men with prostate cancer and 52 urological controls in 2 hospitals in Barcelona found that longer AGD in men with normal in-utero sexual development was related with a lower risk of prostate cancer (41). The results of 2 studies conducted on adult men showed that disconnection of androgen-mediated pathways in utero was related with the risk of prostate cancer (40). Therefore, having a longer AGD ${}_{\text{AS}}$ mention a higher chance of having higher testosterone in adult and, finally, a great risk of suffering a more strict form of prostate cancer (74).

### AGD measurements and PCOS

Studies included in our systematic review showed that AGD measurements were longer in the PCOS group (31, 45). A case-control study of 156 PCOS cases and 180 reproductively healthy women showed that AGD was longer in individuals with PCOS than in the control group (31). A cohort study reported that AGD was more prevalent in newborn daughters of women with PCOS compared with a control group with no PCOS. This study suggested AGD may be a potential marker of the downstream risk of PCOS (44). The evidence confirm earlier study that identified during PCOS, women fetuses may expose higher T levels and suggest that AGD may supply postnatal `read-out' of their prior intrauterine hormonal environment (75).

A cross-sectional study among healthy young women found that AGD was positively associated with the number of ovarian follicles. This relationship was confirmed in women with PCOS, suggesting that high prenatal testosterone levels and follicular growth in PCOS, may have common fetal origins (67). Indeed, androgen exposure in utero increased follicular recruitment in females (76).

Results from a descriptive study applying a retrospective list review of 128 patients aged 12-20, showed that androgen exposure in utero increased serum anti-Müllerian hormone, a marker for PCOS (77). Previous studies revealed that intrauterine androgen exposure increases the length of AGD (78, 79). Therefore, exposures to intrauterine and postnatal androgens are associated with PCOS and may also affect the length of AGD. Finally, AGD may have clinical utility when measuring human fetal androgen levels during pregnancy (43).

### AGD measurements and POP

According to evidences included in the study, significant differences were observed between POP individuals and the controls for AGD ${}_{\text{AC}}$ and AGD ${}_{\text{AF}}$ . In that study, women with POP had longer AGD ${}_{\text{AC}}$ (46). A case-control study among 58 patients showed differences between the AGD ${}_{\text{AF}}$ (which is shorter in cases of prolapse), AGD ${}_{\text{AC}}$ distances, and length of genital hiatus (which is longer in cases) (29). As a result, AGD is presently utilized to quantify the volume of vaginal region hiatus in women with POP since it is less expensive and has a more accessible approach than other methods (80, 81). Hence, AGD may be a more accessible marker for clinical use in calculating the amount of the genital region hiatus in prolapses.

### AGD measurements and fetal gender

Based on the study included in the current systematic review, AGD is a novel biomarker that may play a role in determining fetal sex. Few studies exist about fetal AGD and the differences between females and males. A previous cross-sectional study found that measuring AGD in the first trimester of pregnancy is a novel method for determining fetal sex. In that study, AGD was greater in male fetuses than in female fetuses (49). A previous study of 111 cases with a singleton pregnancy between 11 and 13 wk and 6 days found that when ultrasound detected AGD 4.8 mm, the likelihood of the pregnancy being female increased (11). Some evidence reported that AGD was significantly shorter in females than in males (48, 49). Previous study fetal gender was recognize by ultrasound in 310 singleton pregnancies at 11-14 wk of gestation, explain that a cut-off of 4.8 mm was determined to predict male ( ≥ 4.8 mm) or female ( < 4.8 mm) fetuses (47). These results are in agreement with those of the Fowler study (48) although the cut-off value in this study (47), was 4.8 mm as opposed to 5 mm. This 4.8 mm cut-off demonstrated a high accuracy of AGD in specify male from female fetuses, resulting in sex determination in 87% of the males and 89% of the females. The results of a previous study in 87 term neonates (38 ≥ wk) showed that AGD was twice as common in male infants (average 22 mm) as in female infants (average 11 mm) (82).

### AGD measurement, hypospadias, and cryptorchidism

The results of the current systematic review reported a positive relationship between AGD, hypospadias, and cryptorchidism (50). The findings of the studies included in our study revealed that, when compared to the general population, shorter AGD were statistically significant in fetuses with hypospadias (30). Results of a systematic review and meta-analysis showed that AGD was shorter in boys with hypospadias and cryptorchidism (83). In a large cohort study of boys of pre-pubertal age, AGD was significantly shorter in boys with hypospadias compared to boys with normal genitalia in the healthy control group (52). Another study among boys < 2 yr of age diagnosed with cryptorchidism or isolated hypospadias and recruited from clinics at Cambridge University hospital showed that boys with hypospadias or cryptorchidism had significantly lower AGD and penile length than the healthy control group (53). 2 previous studies have shown the relationship between shortened AGD and cryptorchidism. Also, the latter study reported reduced AGD in boys with hypospadias (51, 84). One study among 52 fetuses suggested that, in the prenatal examination and counseling of male external genital abnormalities, AGD may be used as a supplemental objective sonographic measure (30).

### AGD measurements, fertility and semen parameters

The present study shows that the majority of the studies demonstrated that AGD was related to semen parameters and fertility in men (60). A study among Spanish children aged between 9 and 11 yr, reported that longer AGD was positively associated with increased testicular volume (28). One study among 473 men showed that AGD was related to semen parameters. In that study, longer AGD was related with great sperm concentration, total sperm count, and total motile sperm count (54).

A cross-sectional study among infertile men aged between 25 and 38 yr detected a positive relationship between AGD ${}_{\text{AS}}$ and total sperm count, sperm concentration, and total sperm motile count (55). Similar results in another study conducted in the US on male students (56) and infertile men referred to andrology clinics (58) have been reported. However, study results among Caucasian young men from southern Spain reported that AGD was not related to the semen parameter (22), which is in opposite to the above results (56, 58, 59). However, the reasons for the controversial findings to date are not yet clear, but they could according to differences in studied age ranges, residual confounding, or even ethnic factors.

To our knowledge, the current reports represent the first presentation of the use of assessing AGD in clinical practice to assist patient care. AGD may prognosis normal male genital growth and sperm generation and could therefore provide a new tool to determine reproductive potential in men. Moreover, it may give the professionals additional prognostic information when counseling azoospermic men. Therefore, the results of this systematic review suggested that AGD can help diagnose reproductive function and infertility in men.

### AGD measurements and gynecological related disorders 

The findings of the studies in this systematic review demonstrated the relationship between AGD and gynecological conditions in women (64). A prospective cohort of 300 fertile women showed that, when comparing women with below-average AGD to those with above average AGD, the incidence of encounters owing to gynecological issues was much greater (12). Another cohort study among 119 women suggested measures of AGD as risk factors for episiotomy. That study introduced in the episiotomy group AGD ${}_{\text{AC}}$ was significantly shorter (61). Another study measured AGD in pregnant women (85). Better AGD measurements in women could thus be added to routine gynecological assessment and admission in the delivery room, providing critical data in women at risk for later gynecological morbidities (12). Another study among 1154 Indian infants reported that, compared with infants with descended testes, AGD was significantly shorter in infants with undescended testes (63). A prospective, observational study among 150 adult men aged 18-55 yr showed that in the premature ejaculation group, AGD ${}_{\text{AP}}$ and AGD ${}_{\text{AS}}$ were lower than in the control group (66). Therefore, AGD may be clinically useful as a measure of androgen action during pregnancy (65). Studies have shown that fertility and adult sperm production are related to AGD (56, 58). A cohort study suggested that total motile sperm count and sperm concentration after varicocelectomy were associated with longer AGD in men. However, no relationship was observed between improvements in semen volume or sperm motility and AGD length (62).

### AGD measurements and ovarian follicular number

According to studies included in the present systematic review, poor ovarian response was associated with shorter AGD ${}_{\text{AC}}$ and AGD ${}_{\text{AF}}$ . In that study, AGD ${}_{\text{AC}}$ and AGD ${}_{\text{AF}}$ were positive and significant in relation to the total number of oocytes (33). A cross-sectional study reported that AGD ${}_{\text{AF}}$ was positively associated with ovarian follicle number (67). Some previous studies suggested that AGD length is associated with female reproductive function (57, 67, 71, 86). More studies reported that AGD was longer in women with PCOS (31), and daughters born of these women had longer AGD (78). So, our results may contribute to the claim for using AGD as a marker of the intrauterine hormonal milieu in epidemiological and clinical research.

### AGD measurements, maternal and birth outcome 

The studies included in our systematic review found links between AGD and maternal and infant characteristics. A cross-sectional study among 133 Korean infants reported that AGD1-3 in the low-birth-weight group were significantly lower than newborns in the control group with normal birth weight (68). A previous study showed that AGD in males was significantly associated with birth weight and gestational age. In that study, AGD in females was associated with birth weight, gestational age, and length (70). Another study demonstrated that AGD was longer in a male newborn with higher cord serum free triiodothyronine, free thyroxine, thyroid-stimulating hormone, and a lower thyroxine/triiodothyronine ratio. In that study, the association between AGD and thyroid-stimulating hormone was not statistically significant in the female neonate (69). A feasible practice is that T3 enhance skeletal muscle growth by adding the frequency and dimension of muscle fibers between the anus and the genital near the central perineal tendon (87). Another possibility is that the placenta influences fetal thyroid hormones and AGD (88).

## 4. Conclusion

Using quantitative indicators such as AGD to determine the prognosis and early diagnosis of diseases in the future may be of great help in therapeutic interventions and the treatment of cases in the early stages of the disease. Along the way, we should not neglect variables such as age, gender, and stressful life events that may confound the relationship pathway between AGD and disorders.

## 5. Author's statement

We declare that this manuscript is original, has not been previously published or submitted elsewhere for publication, and is not under consideration by another journal.

##  Conflict of Interest

The authors declare that there is no conflict of interest.
